# Neuronal survival in the brain: neuron type-specific mechanisms

**DOI:** 10.1038/cddis.2017.64

**Published:** 2017-03-02

**Authors:** Ulrich Pfisterer, Konstantin Khodosevich

**Affiliations:** 1 Biotech Research and Innovation Centre (BRIC), University of Copenhagen, Copenhagen, Denmark

## Abstract

Neurogenic regions of mammalian brain produce many more neurons that will eventually survive and reach a mature stage. Developmental cell death affects both embryonically produced immature neurons and those immature neurons that are generated in regions of adult neurogenesis. Removal of substantial numbers of neurons that are not yet completely integrated into the local circuits helps to ensure that maturation and homeostatic function of neuronal networks in the brain proceed correctly. External signals from brain microenvironment together with intrinsic signaling pathways determine whether a particular neuron will die. To accommodate this signaling, immature neurons in the brain express a number of transmembrane factors as well as intracellular signaling molecules that will regulate the cell survival/death decision, and many of these factors cease being expressed upon neuronal maturation. Furthermore, pro-survival factors and intracellular responses depend on the type of neuron and region of the brain. Thus, in addition to some common neuronal pro-survival signaling, different types of neurons possess a variety of 'neuron type-specific' pro-survival constituents that might help them to adapt for survival in a certain brain region. This review focuses on how immature neurons survive during normal and impaired brain development, both in the embryonic/neonatal brain and in brain regions associated with adult neurogenesis, and emphasizes neuron type-specific mechanisms that help to survive for various types of immature neurons. Importantly, we mainly focus on *in vivo* data to describe neuronal survival specifically in the brain, without extrapolating data obtained in the PNS or spinal cord, and thus emphasize the influence of the complex brain environment on neuronal survival during development.

## Facts

- During development neurons express a set of pro-survival/death molecules that are not present in adult brain.
- Neuronal survival in the brain often relies on different external factors in comparison with the spinal cord and PNS.
- Different types of neurons in the brain possess some common, but also distinct components of pro-survival signaling.
- Immature neurons are more vulnerable to stress factors that trigger neuronal death than mature neurons.

## Open questions

- How abundant are distinct components of pro-survival signaling in different types of neurons that might adapt neuronal survival to the region of the brain, that is, neuron type-specific survival?
- How do survival mechanisms of embryonically and adult-born neurons differ, that is, survival in immature *versus* mature brain?
- During what period of brain development do the various types of neurons die?
- What mechanisms account for higher vulnerability of immature neurons to stress factors?

During brain development, an excessive number of neurons is generated and, depending on the region and neuronal type, a varying number of neurons die before they mature.^1, 2, 3, 4, 5^ A high rate of neuronal death also occurs in the regions of adult neurogenesis.^6, 7, 8, 9^ The process of neuronal overproduction and elimination is necessary to optimize brain connectivity. Disturbances in regulating developmental neuronal death not only change cell composition and connectivity within local neuronal networks, but also alter global brain activity and, thus, cognition. Several types of brain disorders enhance the death of immature neurons (i.e., postmitotic neurons, but before complete maturation) during brain development that could lead to decline in cognitive abilities. After maturation, neurons become resistant to the signaling that was involved in the life/death decision at immature stages since, once neurogenesis is halted, it is advantageous to protect mature neurons that cannot be produced again (protection of immature and mature neurons is compared in Benn and Woolf^10^ and Kole *et* *al.*^11^).

**Figure 1:**
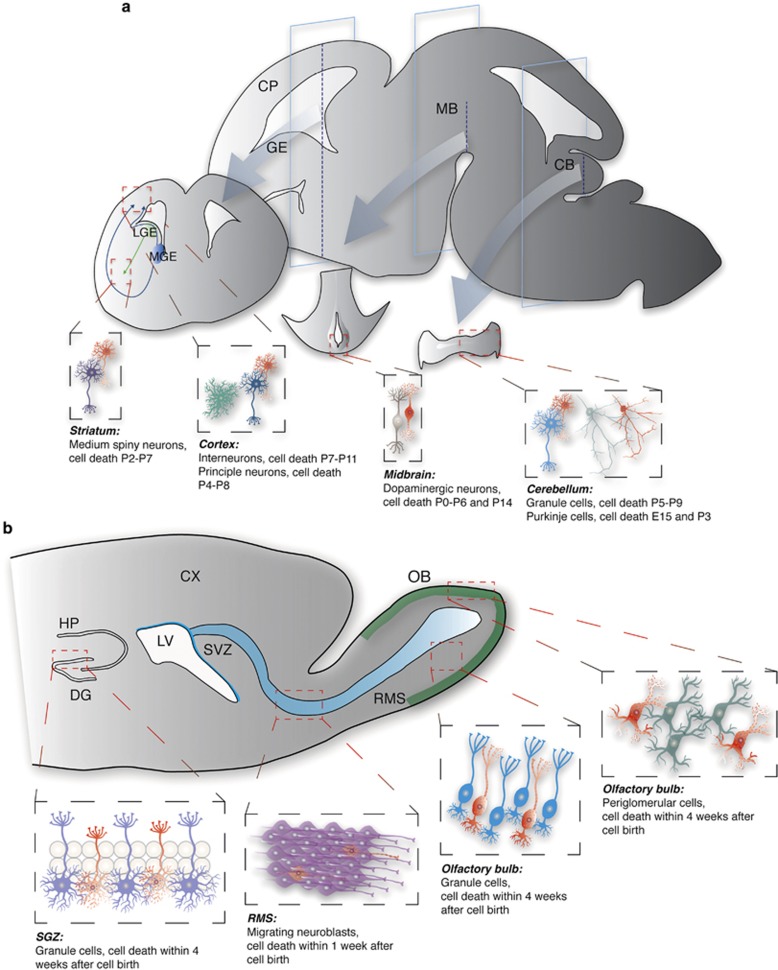
Neuronal death during embryonic and adult neurogenesis. (**a**) During embryonic brain development, neurons are born around the ventricles and migrate toward various brain regions. Cortical principal neurons and interneurons are born in the dorsal and ventral telencephalon, respectively. The majority of interneurons are born in the medial and caudal (data not shown) ganglionic eminences (MGE and CGE, respectively), whereas striatal medium spiny neurons are born in the lateral ganglionic eminence (LGE). Dopaminergic and cerebellar neurons are born in the ventricular zones of the mesencephalon and cerebellum, respectively. Red cells in each region depict dying immature neurons. Peak period of developmental cell death is given for each type of neurons. (**b**) The SGZ of the dentate gyrus in the hippocampus and the SVZ of the lateral ventricles continue to generate neurons throughout life. The SGZ generates neuroblasts that translocate within the SGZ and mature into excitatory granule cells. Neuroblasts that are generated in the SVZ migrate a long distance through the rostral migratory stream toward the olfactory bulb and mature into two major populations of inhibitory interneurons – granule and periglomerular cells. More than half of adult-generated neurons die by apoptosis. Red cells in each region depict dying immature neurons. Peak period of developmental cell death is given for each type of neurons. CB, cerebellum; CP, cortical plate; CX, cortex; DG, dentate gyrus; GE, ganglionic eminence; HP, hippocampus; LGE, lateral GE; LV, lateral ventricle; MB, midbrain; MGE, medial GE; RMS, rostral migratory stream; OB, olfactory bulb

There are two distinct modes of neurogenesis – although the majority of neurons are generated during the embryonic period and their production is discontinued either in the embryonic brain or early postnatally (later referred to as embryonic neurogenesis),^12^ some populations of neurons are continuously generated throughout the life of an animal (later referred to as adult neurogenesis)^13, 14^ (see Figures 1a and b, respectively). The death of neurons that are born embryonically reaches a peak in the neonatal brain and affects neurons that are still immature,^15, 16, 17^ and the critical period for survival of adult-generated neurons is within 4 weeks after their birth; following this period of maturation, they become resistant to cell death.^8, 9, 18^

**Figure 2:**
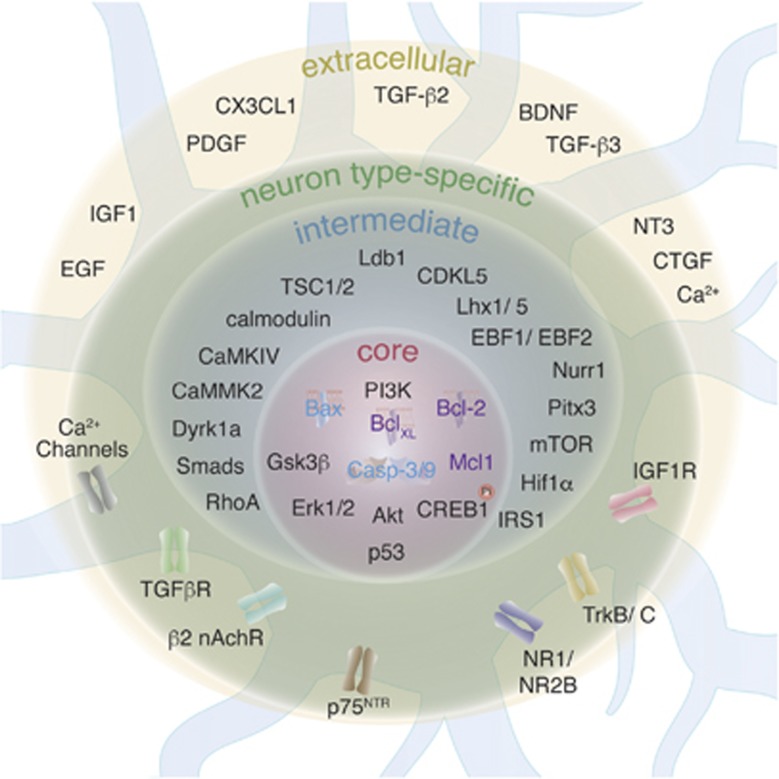
Components of survival/death signaling in immature neurons. Extracellular pro-survival factors that are available in a certain brain area stimulate a variety of receptors and ion channels on neurons located in the area. Transcription factors involved in neuronal differentiation determine what combination of receptors and ion channels will be expressed on a particular neuron. Such neuron type-specific combination of receptors and channels propagates pro-survival signaling to intermediate components, some of which express broadly, whereas others have restricted expression only in one or few types of neurons. Finally, all pro-survival signaling converges on core components that inhibit neuronal death

Principles of neuronal survival are often generalized and data from different areas of the CNS are extrapolated to the CNS as a whole. Indeed, pro-survival signaling does converge on some common core components (Figure 2). However, data accumulated over the recent years show that different types of neurons in the brain might use different pro-survival mechanisms as there are a variety of routes by which core pro-survival components could be activated. Thus, we propose 'neuron type-specific' pro-survival mechanisms that will heavily rely upon (1) composition of extracellular pro-survival factors that are available in a certain brain area at a certain time period, (2) composition of transmembrane molecules (e.g. receptors or ion channels) that are expressed on distinct types of neurons and (3) composition of cytosolic molecules that could propagate pro-survival signaling from the cell membrane toward common core components (Figure 2).

It should be noted that not only pro-survival, but also pro-death pathways could be neuron type specific. In general terms, it is pro-survival signaling that blocks intrinsic pro-death signaling, and when there is a lack of pro-survival signaling, pro-death pathways are triggered. However, in a recent paper^19^ it was shown that survival of CNS neurons during development is regulated by 'dependence receptors' that activate pro-death signaling when not bound to their ligands (reviewed in Dekkers *et al.*^20^). Although the extent of expression and the number of dependence receptors still remain to be determined in the developing brain, the presence of such a mechanism indicates that neuron type-specific pro-death pathways do exist.

## Neuron type-specific pro-survival mechanisms

As different types of neurons survive in different brain areas and at different periods of brain development, the transcriptome of the surviving neuron should 'prepare' the neuron to survive in a certain environment. The preparation is coordinated by distinct sets of transcription factors that are involved in differentiation of specific types of neurons. These transcription factors drive expression of transmembrane and intracellular molecules that are necessary to recognize and respond to the local environment. Neurons failing to differentiate properly are less likely to respond to signals from local brain environment and could be eliminated during maturation. Interestingly, the period of developmental cell death differs across types of neurons/brain areas. For instance, GABAergic interneurons of the cortex and medium spiny neurons exhibit one peak of cell death at P7-P11^1^ and P2-P7,^21^ respectively, whereas two distinct peaks of developmental cell death have been observed for dopaminergic neurons, at P0-P6 and ~P14,^2^ and for Purkinje cells, at ~E15 and ~P3.^22^

The difference in survival mechanisms between embryonically and adult-born neurons illustrates the importance of time period of neuronal survival with regard to brain maturation, since embryonically born *immature* neurons must survive in *immature* brain, whereas adult-born *immature* neurons must survive in *mature* brain. Thus, there is high pressure for adult-born neurons to integrate into the pre-existing mature circuits, which is absent for embryonically born neurons. This is supported, for instance, by a higher vulnerability of adult-born neurons to impairment in NMDA receptor (NMDAR) expression, since ablation of NR1 or NR2B subunit markedly augments death of adult-born neurons during maturation,^23, 24, 25^ whereas studies of global or early postnatal knockout of these subunits do not report increase in apoptosis of embryonically produced neurons.^26, 27, 28^

The effect of brain maturation on neuronal survival might also be illustrated by a decrease in survival of small axonless neurons – a type of neurons that is generated both during embryonic and adult neurogenesis.^29^ The majority of these neurons survive in the deep cortical layers when circuits are still immature, and gradual maturation of the brain correlates with a decreased number of newly added neurons,^29^ although the number of these neurons could be increased by pathological conditions such as stroke.^30^

Support of neuronal survival by the local environment depends on whether a specific factor itself and its receptor are expressed in the region. Availability of pro-survival factors varies within the brain and even cortical layers,^31, 32, 33^ and response to different pro-survival factors markedly changes over a course of neuronal maturation.^34^ Moreover, certain intracellular pro-survival molecules are present only in some types of neurons, but not in others. For instance, BDNF promotes survival of dopaminergic neurons, medium spiny neurons and cerebellar granule cells,^35, 36, 37^ but it is dispensable for survival of GABAergic neurons in the cortex^1^ although the latter express TrkB receptor and BDNF is available in the surrounding environment.^31, 38^

In the following, we summarize the evidence for neuron type-specific pro-survival mechanisms during embryonic and adult neurogenesis (see overview in Table 1).

### Embryonic neurogenesis: glutamatergic neurons

**Figure 3:**
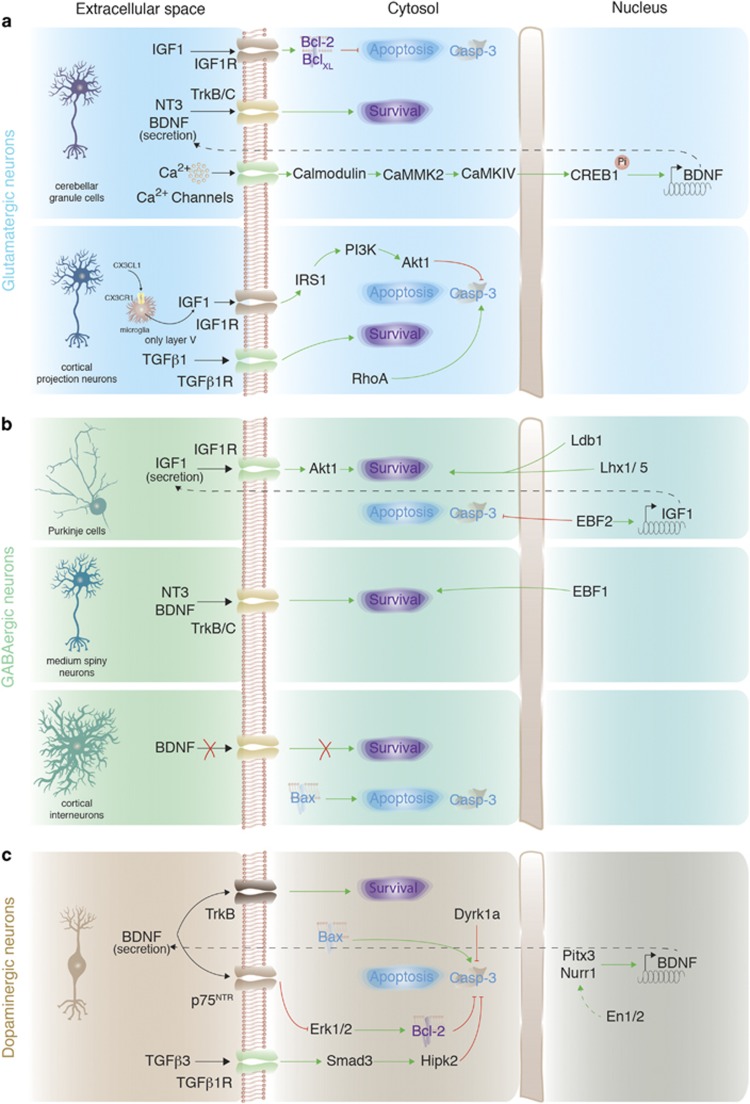
Neuron type-specific pro-survival signaling in embryonically born neurons. (**a**) Signaling involved in survival and cell death of glutamatergic neurons exemplified by cerebellar granule cells and cortical projection neurons. (**b**) Pro-survival and apoptotic signaling in GABAergic neurons illustrated by Purkinje cells, medium spiny neurons and cortical interneurons. (**c**) Signaling regulating survival or cell death in dopaminergic neurons. Green arrows: activation of signaling; dashed green arrow: proposed activation of signaling; red blunt arrows: inhibition of signaling; black arrows: activation of receptors on immature neurons from the extracellular space; dashed black arrows: protein secretion to the extracellular space; red cross: lack of signaling; Pi: phosphorylation

The most information regarding survival of glutamatergic neurons in the brain was obtained by studying cerebellar granule cells and principal neurons of the hippocampus and cortex (Figure 3a). The peak of cortical principal neuron cell death is at P4–P8,^39^ whereas the majority of immature cerebellar granule cells die at P5–P9.^40^ Although knockout of a single neurotrophic factor or its receptor does not have large effects on neuronal survival during brain development,^41^ double knockout of *Ntrk2* and *Ntrk3* (genes coding for TrkB and TrkC, respectively) results in the massive death of immature granule cells in the cerebellum and dentate gyrus.^42^ This could be explained either by redundancy of intracellular pro-survival pathways that are triggered by each of the receptors or by compensatory effects in knockout mice. Furthermore, often data obtained *in vivo* differs from *in vitro* experiments, highlighting importance of brain environment for action of a particular pro-survival factor. For instance, BDNF was shown to promote neuronal survival in the culture,^43^ but deletion of *Bdnf* in all postmitotic neurons in the brain did not have a large effect on their survival.^44^

Granule cells of the cerebellum represent a population of glutamatergic neurons that could be a target of pro-survival action of BDNF. Deleting *Camk4* and *Camkk2* genes in mice enhances apoptosis in immature granule cells in the cerebellum, which is associated with a decrease in levels of CREB1 and BDNF expression.^37^ It was proposed that Ca^2+^ entering immature granule cells triggers activation of the calmodulin/CaMKK2/CaMKIV cascade, which, in turn, activates CREB1 and transcription of *Bdnf* gene.^37^ Survival of granule cells is also promoted by IGF1 that enhances expression of Bcl-2 and Bcl-x_L_ thus inhibiting caspase-3 activity.^45^

The existence of neuron type-specific pro-survival mechanisms in glutamatergic neurons was recently highlighted by the identification of a pro-survival pathway that was largely restricted to cortical principal neurons of layer V, which require trophic support from microglia to survive during early postnatal development.^46^ Microglia secrete IGF1, which binds to IGF1R on immature layer V neurons and activates the IRS1/PI3K/Akt1 cascade inhibiting caspase-3-dependent apoptosis.^46^ Microglia are activated via CX3CL1, which is released from layer V neurons and interacts with CX3CR1 on microglia.

Interestingly, caspase-3-dependent apoptosis of cortical excitatory, but not inhibitory, neurons was shown to be activated by Rho GTPase RhoA.^47^ Inhibiting RhoA signaling in the developing brain rescues up to 25% of cortical neurons from apoptosis.

### Embryonic neurogenesis: GABAergic neurons

Only few studies have investigated developmental death of GABAergic neurons, and these were mainly focused on Purkinje cells of the cerebellum and medium spiny neurons of the striatum that exhibit a peak of cell death at ~E15 and ~P3,^22^ and at P2–P7,^21^ respectively (Figure 3b). Lhx1/Lhx5 transcription factors together with their co-activator Ldb1 promote survival of postmitotic Purkinje cells at E13.5–E15.5.^48^ Interestingly, two members of the EBF (early B-cell factor) family of transcription factors – EBF1 and EBF2 – are involved in survival of medium spiny^49^ and Purkinje neurons,^50^ respectively, during perinatal development. In Purkinje cells, EBF2 binds to *Igf1* promoter and activates *Igf1* expression that results in local IGF1 secretion and potentiation of Akt1-dependent pro-survival signaling.^51^ All the aforementioned transcription factors were also shown to be involved in differentiation and/or migration of medium spiny and Purkinje neurons, and thus immature neurons might die because they are not able to complete their differentiation programs.

Although, overall, neurotrophins do not have a large role in survival of immature GABAergic neurons, BDNF and NT-3 were shown to enhance survival of immature medium spiny neurons, as they are secreted by midbrain dopaminergic neurons during a critical period of striatal neuron survival and activate pro-survival signaling via TrkB and TrkC receptors.^35^

Recently, it was shown that around 40% of immature cortical GABAergic interneurons die during the first two postnatal weeks (with the peak at P7–P11).^1^ Their survival did not depend on TrkB expression, but was regulated by either cell-autonomous or population-autonomous mechanisms that activated pro-apoptotic Bax signaling.

### Embryonic neurogenesis: dopaminergic neurons

Apoptosis of immature dopaminergic neurons occurs at two developmental stages – at P0–P6 and ~P14.^2^ Three main transcription factors involved in specification dopaminergic neurons – *Nurr1*, *Pitx3* and *En1* – also regulate their survival.^52, 53, 54, 55^ Both Nurr1 and Pitx3 were shown to activate expression of BDNF,^56, 57^ which promotes survival of a subpopulation of dopaminergic neurons from E16 onward^36^ via TrkB receptors^58, 59^ (Figure 3c).

Another BDNF receptor, low-affinity neurotrophin receptor p75^NTR^, promotes cell death of immature dopaminergic neurons.^60^ Expression of p75^NTR^ is repressed by En1/2,^60^ and as En1 was also proposed to co-activate expression of Nurr1-dependent genes,^61^ En1 could enhance survival of immature dopaminergic neurons via two pathways – enhancing BDNF expression (via Nurr1) and repressing p75^NTR^ expression. Pro-death signaling from p75^NTR^ suppresses ERK1/2 activity and likely inhibits anti-apoptotic activity of Bcl-2 family members,^60^ thus activating a classical apoptosis pathway via Bax, caspase-3 and caspase-9.^62^ Caspase-3/-9 activation is inhibited by dual-specificity tyrosine-phosphorylation regulated kinase 1A (Dyrk1a), a Down syndrome-associated gene.^63^

Involvement of neuron type-specific signaling in survival of dopaminergic neurons is highlighted by inhibition of developmental apoptosis by TGF*β*-Smad-Hipk2 pathway.^64^ Interestingly, although transforming growth factor (TGF) *β*1 and *β*2 had little effect on modulation of survival of immature dopaminergic neurons, stimulation by TGF*β*3 led to activation of Smad2/3 that directly interacted with Hipk2 and inhibited caspase-3-dependent apoptosis.

### Adult neurogenesis: subventricular zone (SVZ)

**Figure 4:**
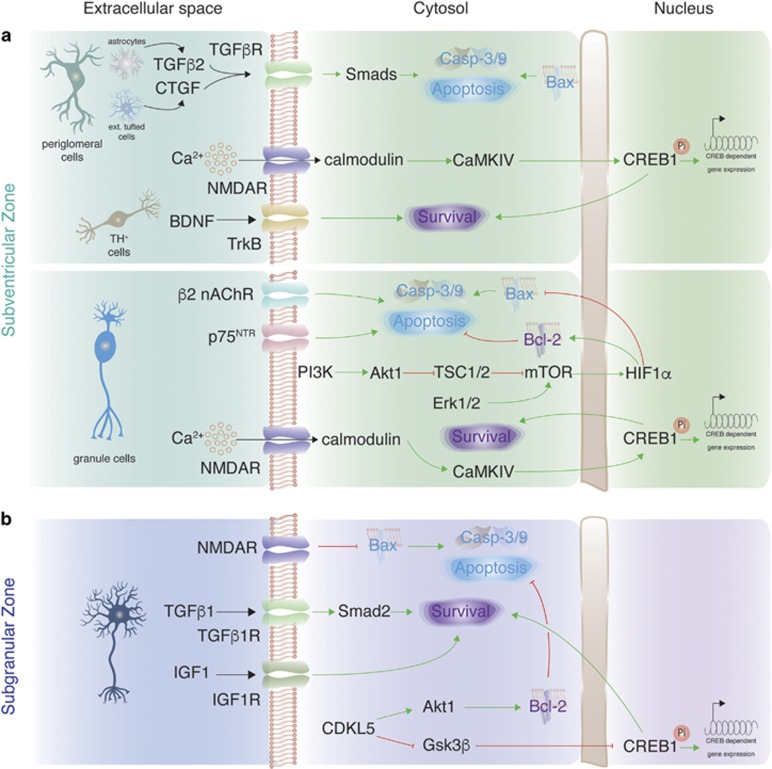
Neuron type-specific pro-survival signaling in adult-born neurons. (**a**) Signaling involved in survival and cell death of immature neurons that are born during adult neurogenesis in the SVZ. (**b**) Signaling involved in survival and cell death of immature neurons that are born during adult neurogenesis in the SGZ. Green arrows: activation of signaling; red blunt arrows: inhibition of signaling; black arrows: activation of receptors on immature neurons from the extracellular space; Pi: phosphorylation

Survival of postnatally born neurons in the olfactory bulb is regulated by neuronal activity (Figure 4a). Ablation or enhancement of olfactory activity onto maturing granule cells decreases or increases their survival, respectively.^65, 66^ However, similar enhancement does not affect periglomerular neurons,^9, 66^ which could be explained by neuron type-specific pro-survival mechanisms. Furthermore, stimulation of periglomerular neurons by a single odorant decreases their survival in the region that is activated by the odorant.^9^ Apoptosis is stimulated by connective tissue growth factor (CTGF) that, in combination with TGF*β*2, activate TGF*β*Rs and Smads in immature periglomerular neurons.^9^

Few neurotransmitter receptors on newborn SVZ neurons mediate pro-survival effects of neuronal activation. Glutamate NMDAR activity is required for survival of neuroblasts during their migration from the SVZ through the RMS and when maturing in the olfactory bulb.^23, 67^ This pro-survival effect likely depends on Ca^2+^ that enters into neuroblasts via NMDAR. When already in the olfactory bulb, expression of nicotinic acetylcholine receptor (nAChR) subunit *β*2 regulates apoptosis in newborn granule cells.^68^ Knockout of the subunit results in 50% increase in survival of immature neurons, and stimulation of nAChR could be considered as another 'negative' regulator of immature neuronal survival in postnatal neurogenesis, similar to CTGF.

Phosphorylation of CREB1 was shown to promote survival of SVZ-derived neuroblasts,^69, 70^ where CREB1 might be activated by Ca^2+^ signaling via calmodulin and CaMKIV.^71, 72^ As NMDAR are involved in survival of SVZ neuroblasts,^23, 67^ and upon opening they allow Ca^2+^ entry into neuroblasts,^67^ it is likely that Ca^2+^ entry via NMDAR triggers CREB1-dependent pro-survival cascade (although other receptors on neuroblasts could also mediate Ca^2+^ entry).^72, 73^ Knockout of *Creb1* was shown to decrease expression of the polysialylated isoform of the neural cell adhesion molecule (PSA-NCAM),^70^ which, in turn, could promote survival of immature olfactory bulb neurons by inhibiting p75^NTR^ expression.^74^ Among p75^NTR^ activating neurotrophins only the role of BDNF in postnatal SVZ neurogenesis has been studied, and *Ntrk2* knockout decreases the survival of dopaminergic periglomerular neurons, but not any other cells.^75, 76^

Mammalian target of rapamycin (mTOR) pathway promotes the survival of SVZ neuroblasts via hypoxia-inducible factor 1a (HIF1A).^77^ Tuberous sclerosis proteins 1 and 2 (TSC1/2) inhibit mTOR, and HIF1A is strongly upregulated in *Tsc1−/−* neuroblasts, thereby increasing their survival.^77^ mTOR is most likely activated by PI3K/Akt1 signaling as many components of this pathway were shown to be present in SVZ neuroblasts.^72, 78^

Finally, pro-survival signaling in newborn SVZ neurons converges on Bcl-2 family members and caspase−3/−9.^7, 79^

### Adult neurogenesis: subgranular zone (SGZ)

Less is known regarding neuronal survival in the SGZ in comparison with the SVZ. Activation of NMDAR on newborn SGZ neurons enhances their survival,^24^ and it is likely that the pro-survival effect depends on Bcl-2 stimulation (Figure 4b).^80^ Protection of newborn dentate gyrus neurons by Bcl-2 signaling was also shown in transgenic mice that overexpress Bcl-2.^81^ Bcl-2 activity might be stimulated by Akt1 signaling, which was shown to enhance neuronal survival in the SGZ.^82^ Cyclin-dependent kinase-like 5 (CDKL5) activates Akt1 and also inhibits Gsk-3*β* thus activating CREB1-dependent gene expression. Similar to the SVZ, apoptosis in newborn SGZ neurons converges on Bcl-2/Bax activity.^6^

Two growth factors promote survival of granule cells in the SGZ – TGF*β*1 and IGF1.^83, 84^ Importantly, both factors have little (if any) contribution to survival of adult-born neurons in the olfactory bulb,^9, 85^ indicating neuron type-specific role of TGF*β*1 and IGF1 in survival of adult-born neurons.

## Common signaling that regulates neuronal survival in the brain

Many neuron type-specific pro-survival pathways eventually converge on pro-apoptotic and pro-survival members of Bcl-2 family and caspase-3/caspase-9 (Figure 2). Neuronal apoptosis in the brain is inhibited by Bcl-2 and Bcl-x_L_ pro-survival proteins,^86, 87, 88, 89^ whereas pro-apoptotic proteins, mainly Bax and Bak, promote neuronal death.^87, 88^ Massive death of immature neurons in the brain of *Bcl2l1−/−* (gene name for Bcl-x_L_) mice suggests that Bcl-x_L_ is the major neuronal pro-survival protein of Bcl-2 family,^86, 87^ and it becomes important for survival only at the stage of postmitotic neurons, but not before.^88^ Another anti-apoptotic member of the Bcl-2 family, myeloid cell leukemia 1 (Mcl-1), was also shown to be critical for survival of immature neurons during embryonic development.^90^

Several transcription factors promote neuronal survival, most likely by activating transcription of pro-survival genes and/or inhibiting pro-apoptotic genes. A family of myocyte enhancer factor 2 (MEF2) transcription factors, MEF2A, 2C and 2D, are expressed in the mouse brain during development and are critical for the survival of immature neurons.^91^ Widespread loss of neurons was also reported for knockout of another transcription factor – p73 (a member of p53 family proteins).^92^ The loss of neurons started to be visible during second postnatal week, and was attributed to the anti-apoptotic role of the truncated form of p73, ΔNp73, which antagonizes p53 function and inhibits Bax and caspase-3/-9-dependent apoptosis.^93^ Finally, members of the CREB family of transcription factors, CREB1 and CREM, activate pro-survival signaling in postmitotic neurons around the time of perinatal development (E16.5-P0).^94^

Activity-dependent survival of immature neurons via action of GABA and/or glutamate neurotransmitters was proposed for many neuronal subtypes.^95^ For instance, deletion of syntaxin-binding protein 1 (*Stxbp1*) that is required for synaptogenesis and neurotransmission results in widespread neuronal death during brain development.^96^ Furthermore, pharmacological inhibition of NMDAR leads to a pronounced decrease in survival of neurons during postnatal brain development.^97, 98, 99^ However, as discussed above, knockouts of genes coding for NMDAR subunits show marked increase in neuronal death only during adult neurogenesis.^26, 27, 28, 100^

Neuronal activity also generates reactive oxygen species (ROS) that could damage maturing neurons and trigger apoptosis. Protection from ROS is particularly important for immature neurons since they are often easier to excite than mature ones.^101, 102^ It was recently shown that knockout of the gene coding for the antioxidant protein lanthionine synthetase C-like protein 1 (LanCL1) causes massive neuronal death in the brain due to reduced glutathione-mediated antioxidant defense and via Bax activation.^103^

## Survival of neurons in injured brain

Immature neurons are more vulnerable to stress factors than mature neurons, as it is easier for external stimuli to trigger neuronal death during development than in adult brain.^11^ Although the exact mechanisms of such vulnerability are unknown, it is likely that neurons over maturation devise a highly protective strategy against any external stress. Furthermore, expression of some pro-death molecules, for example, dependence receptors,^19, 20^ could be limited to immature neurons. Therefore, similar stress factors might be more potent enhancers of neuronal death during development than in adult brain.

In addition to common stress factors that stimulate neuronal death both during development and in adult, few factors are specific for the developing brain – for instance, misplacement of neurons could trigger their death due to impairment in neuronal connectivity. Certain types of immature neurons are more strongly affected by the stress than the others highlighting neuron type-specific mechanisms of survival. Below we discuss factors that affect survival of neurons during abnormal brain development.

### Oxidative stress

**Figure 5:**
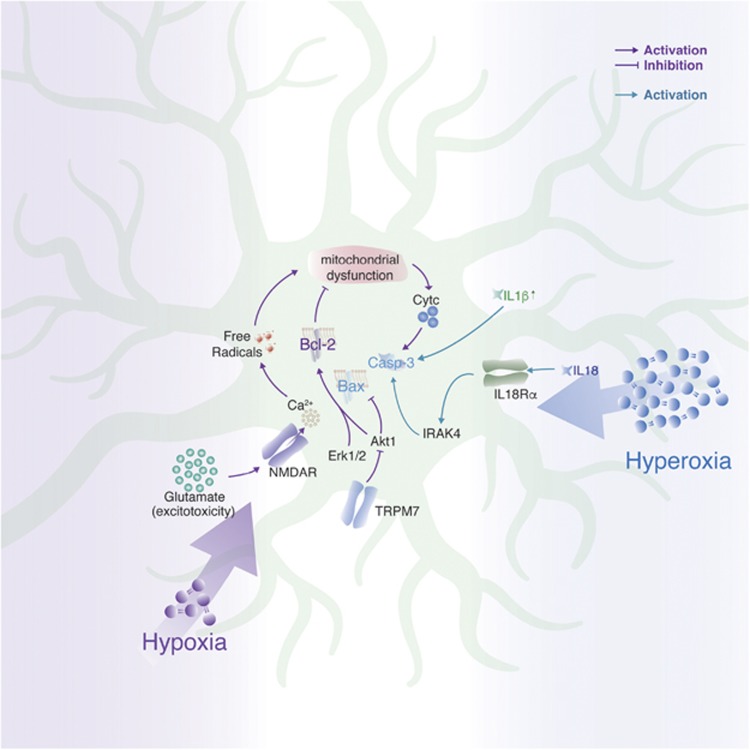
Cell signaling under hypoxic (purple arrows) and hyperoxic (blue arrows) conditions in immature neurons *in vivo*. Arrows: activation of signaling; blunt arrows: inhibition of signaling; vertical small arrow: elevated expression level

Oxidative stress contributes to severe neurodevelopmental deficits in the developing mammalian brain caused by chronic exposure to either reduced (hypoxia–ischemia) or elevated (hyperoxia) levels of oxygen (Figure 5).

Perinatal hypoxia–ischemia or neonatal stroke is the main cause of neurodevelopmental deficits in newborns. It is accompanied by an overall decrease in cortical and hippocampal volumes due to neuronal death and atrophy. One of the major causes of neuronal death is excitotoxicity due to overactivation of NMDAR on immature neurons by the release of glutamate.^104, 105^ Pathological influx of Ca^2+^ via NMDAR is followed by aberrant production of free radicals and mitochondrial dysfunction, which leads to the release of cytochrome C and, consequently, neuronal death.^106, 107^ Importantly, interneurons were shown to be less susceptible to hypoxic cell death – although neonatal hypoxia slows maturation of interneurons, it does not affect their survival.^108^

A glutamate-independent mechanism contributing to hypoxia–ischemia-induced neuronal death reveals transient receptor potential melastatin 7 (TRPM7) as a key factor.^109^ As early as 24 h after neonatal ischemic insult, TRPM7 protein levels were upregulated, which might lead to increase in caspase-3-dependent apoptosis by inhibiting Akt1 and promoting Bax *versus* Bcl-2 expression.

Overexposure to oxygen could cause hyperoxia in the brain, which was shown to affect preterm born neonates receiving oxygen supplementation.^110^ Hyperoxia mainly affects cortical areas and in mice the effect on neuronal survival is most pronounced between P3 and P7.^111^ Apoptosis is caspase-3 dependent and could be enhanced because of decreased pro-survival signaling from Akt1 and Erk1/2.^112^ Importantly, the effect is limited to immature neurons, as hyperoxia at later ages does not affect neuronal survival. Hyperoxia also triggers an inflammatory response that could further promote neuronal death via increased levels of several interleukins - IL-1*β*, IL-18 and IL-18 receptor *α* (IL-18R*α*).^113^

### Fetal alcohol spectrum disorders (FASDs)

**Figure 6:**
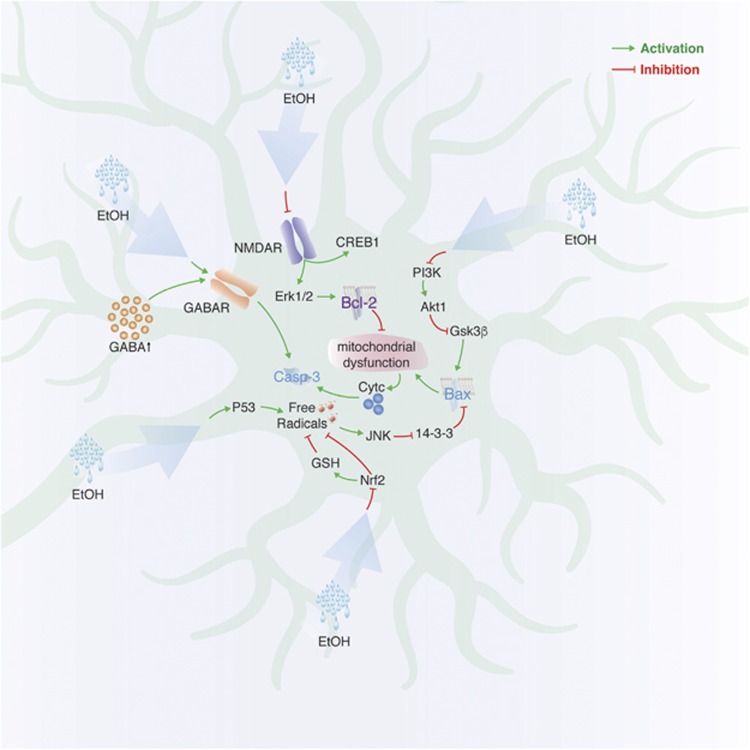
Cell signaling upon alcohol exposure of immature neurons *in vivo*. Green arrows: activation of signaling; red blunt arrows: inhibition of signaling; vertical small arrow: elevated expression level

FASDs are triggered by gestational alcohol exposure and lead to impaired brain development accompanied by deficits in cognitive functions.^114^ Data from animal models of prenatal alcohol exposure suggest that neuronal cell death is one of the major effects contributing to the disease phenotype (Figure 6).^115^

Early postnatal (P7) exposure of rats to EtOH induces widespread apoptosis, indicated by increased activation of caspase-3 as early as 8 h and neurodegeneration within less than 24 h after EtOH treatment.^116^ Differential susceptibility of immature neurons to alcohol-induced stress is underlined by variability of the extent of neuronal death in different brain regions. Thus, the retrosplenial cortex and hippocampus were most affected, whereas the olfactory bulb and piriform cortex exhibited much less apoptosis.^116^ In another study, the overall architecture of mouse brains exposed to alcohol at P7 appeared to be unaltered, but the number of calretinin-positive and parvalbumin-positive GABAergic neurons was strongly reduced, indicating that they are more prone to alcohol-induced cell death when immature.^117^ Misplacing GABAergic neurons could contribute to their death since low doses of prenatal alcohol increase ambient GABA levels in the extracellular space and upregulate GABA_A_ receptors on neuroblasts that lead to aberrant neuroblast migration.^118^

Ethanol possesses NMDA antagonist and GABA_A_ agonist activities and both activities could induce apoptosis during brain development.^97, 119^ Thus, apoptotic effects of ethanol exposure are closely related to those observed with either disrupted NMDA or elevated GABA signaling. The former has been extensively studied in immature neurons using NMDAR inhibitors causing rapid neuronal death of both excitatory and inhibitory neurons associated with decreased Bcl-2, Erk1/2 and CREB1 and increased activated caspase-3 levels.^120, 121, 122^

Embryonically administered EtOH was also shown to decrease activation of pro-survival PI3K/Akt1 signaling and increase activation of glycogen synthase kinase-3*β* (GSK-3*β*).^123^ The latter could stimulate neuronal death by activating Bax and, thus, caspase-3-dependent apoptosis.^124^

Neuronal cell loss as a consequence of alcohol exposure in models of FASD can be attributed in part to oxidative stress. Analysis of the cerebella of P1 rats chronically exposed to ethanol from E6 shows a decrease in mRNA levels of mitochondrial respiration complex genes in granule cells, combined with increased expression of pro-apoptotic p53 and oxidative stress markers.^125^ EtOH also inhibits nuclear translocation of nuclear factor erythroid 2-related factor 2 (Nrf2), a transcription factor that is responsible for expression of those genes that protect against oxidative stress and reduce production of ROS.^126^ In the cerebellum, ROS can activate c-jun N-terminal kinase (JNK) at P4, but not at P7 rats, highlighting a time window in immature granule cells when they are most vulnerable to the oxidative stress.^127, 128^ JNK, in turn, removes pro-survival 14-3-3 protein from its dimer with Bax, thus making it possible for cytosolic Bax to translocate into the mitochondria leading to mitochondrial dysfunction and neuronal apoptosis via release of cytochrome C.

### Traumatic brain injury (TBI)

Although brain injury due to physical trauma is common in both developing and adult brains, the effect of such injury on the immature brain is much more devastating.^129^ Strikingly, in a rat model of the disorder, the extent of neuronal apoptosis is age-related, with the P3–P7 brains being most vulnerable.^130^ Apoptosis of immature neurons was associated with enhanced expression of c-Jun and reduced expression of Bcl-2 and Bcl-x_L_ leading to the release of cytochrome C and neuronal cell death.^130, 131^ Caspase-1 was shown to promote neuronal death by activating two proinflammatory cytokines, IL-1*β* and IL-18, acting via IL-18 R on neurons.^113, 132^ Interestingly, immature neurons are also the most affected by TBI population in the regions of adult neurogenesis in mice.^133, 134^

### Other diseases

Neuronal death contributes to phenotypic effects observed in several other neurodevelopmental disorders. Defects in microtubules because of mutations in tubulin alpha or beta genes are often associated with cortical malformations (e.g., lissencephaly or polymicrogyria) because of neuronal misplacement and subsequent death of misplaced neurons.^135^ For instance, deletion of *Tubb2* gene during brain development causes aberrant neuronal migration and arrest of cells near the ventricles that eventually leads to enhanced neuronal apoptosis.^135, 136^

Although apoptosis was proposed to be augmented in a variety of psychiatric disorders, including schizophrenia and autism spectrum disorders (ASDs), the data were often obtained by analyzing adult brains. Experimental evidence in younger brains is rather limited to gene expression measurements using western blot or PCR.^137^ Furthermore, it remains to be investigated whether a reduction in the number of GABAergic neurons that was reported in postmortem brains of patients with schizophrenia, bipolar disorder and ASDs^138, 139^ occurs before neuronal maturation is finished. In addition, it might be that the strength of marker expression rather than the number of neurons is affected.^140^ Although knockout/knockdown of genes that are associated with psychiatric disorders has been reported to decrease the number of immature neurons in mice,^141^ other studies showed that maturation rather than survival of immature neurons is affected.^142, 143, 144^

## Conclusions

The mammalian brain is the most complex tissue and includes many more neuronal subtypes than other parts of the nervous system. During perinatal development and in the regions of adult neurogenesis, neurons in the brain are overproduced and multitudes of immature neurons die before they reach maturity. Although there are certain core components of survival/apoptotic machinery in immature neurons, it seems that various types of neurons also exploit pro-survival pathways that are specific only for one or few type(s) and not utilized in others. Such *neuron type-specific* components of pro-survival signaling could indicate adaptation toward an optimal survival rate of overproduced neurons according to type of neuron and brain region. The number, type and position of neurons that survived should affect both local neuronal circuits and higher brain activities, for example, oscillations. As there is increasing evidence that some types of neurons are more susceptible to certain injuries in the developing brain, more targeted therapeutic strategies might be needed to treat such brain disorders. The advantage of targeting neuron type-specific pro-survival pathways is to avoid side effects of the therapy on other neuron/cell types that are not affected in the disorder. Future studies will determine the extent to which neuron type-specific pro-survival signaling is utilized in normal brain and in pathological conditions and how it contributes to brain information processing.

## Figures and Tables

**Table 1:** Examples of neuron type-specific pro-survival genes

**Factors**^a^	**Embryonic**						**Adult**			**References**
	**CX EX**	**CB GC**	**MB DA**	**CB PC**	**ST MS**	**CX IN**	**OB PG**	**OB GC**	**DG GC**	
BDNF/TrkB^b^	-	+	+	-	+	-	+^c^	-	?^d^	^35, 36, 37, 44, 56, 58, 59, 76, 145, 146^
NGF/TrkA^e^	-	-	-	-	-	-	-	-	?^f^	^147, 148, 149, 150, 151^
NT-3/TrkC	-	+	-	-	+	-g	?	?	-	^35, 42, 152, 153, 154, 155^
IGF1/ IGF1R	+^h^	+	-	+	?	-i	-	-	+	^45, 46, 51, 84, 85, 156, 157^
*β*2 nAChR	-	?	?	?	-	-g	-	+	-	^68, 158, 159, 160^
TGF*β*/TGF*β*R	+	?^j^	+	?	?	?	+	-	+	^9,64,83,161^
CTGF	-	-	-	-	-	-	+	-	-	^9, 162^
p75^NTR^	-	-	+	-	?	?	+^k^	+	?	^60, 74, 163, 164, 165, 166, 167^
NR1l,,^m^	-	-	-	-	-	-	+	+	+	^23,24,26,100,168,169^
NR2B^l^	-	-	-	-	-	-	+	+	?	^25, 27, 28^

Abbreviations: Neuron types: CX EX, cortical excitatory neurons; CB GC, cerebellar granule cells; MB DA, midbrain dopaminergic; CB PC, cerebellar Purkinje cells; ST MS, striatal medium spiny neurons; CX IN, cortical interneurons; OB PG, olfactory bulb periglomerular cells; OB GC, olfactory bulb granule cells; DG GC, dentate gyrus granule cells

'+' involved; '-' studied, and no involvement was found; '?' no *in vivo* data/controversial data

a Note that only data from *in vivo* experiments are included in the Table, as neuron type-specific mechanisms of survival rely on complex brain environment

b Although TrkB can be also activated by NT-3 and NT-4, usually the phenotype of mice with ablated/disturbed TrkB expression correlates with those mice where Bdnf expression was ablated/disturbed

c Only dopaminergic

d Using different mouse models and experimental conditions, BDNF/TrkB signaling was either shown to be involved in or to be dispensable for apoptosis

e Owing to highly specific expression of TrkA in cholinergic (ChAT+) neurons, none but a subpopulation of ChAT+ neurons in the brain depends on NGF/TrkA, highlighting neuron type-specific pro-survival mechanisms

f Only exogenously delivered NGF was shown to have an effect on hippocampal neurogenesis

g Few gene ablation studies showed lack of effect on neuronal death in the cortex, although interneurons as a subclass of neurons were not studied

h Mainly layer V

i Not all subtypes were analyzed

j Although there are a number of *in vitro* studies showing both positive and negative involvement of TGF*β* in survival of granule cells, there is a lack of *in vivo* data supporting a role of TGF*β* in neuronal survival in the cerebellum

k Likely to be affected as many migrating neuroblasts exhibit p75^NTR^-dependent apoptosis

l Only *in vivo* data regarding involvement of *Grin1* (NR1) and *Grin2b* (NR2B) genes are included in the Table, and studies with pharmacological inhibition of NMDA receptors are omitted

m Knockout of *Grin1* was shown to increase death only in the thalamus,^169^ but not in any other region of the brain, highlighting neuron type-specific pro-survival mechanisms
